# Development of Vitamin D Toxicity from Overcorrection of Vitamin D Deficiency: A Review of Case Reports

**DOI:** 10.3390/nu10080953

**Published:** 2018-07-24

**Authors:** Kornelia Galior, Stefan Grebe, Ravinder Singh

**Affiliations:** Department of Laboratory Medicine and Pathology, Mayo Clinic, Rochester, MN 55905, USA; Galior.Kornelia@mayo.edu (K.G.); Grebe.Stefan@mayo.edu (S.G.)

**Keywords:** vitamin D toxicity, vitamin D overdose, hypercalcemia

## Abstract

Over the past two decades, vitamin D level measurements have become some of the most frequently ordered tests in the laboratory. This increase is due to a growing awareness of widespread vitamin D deficiency and scientific data suggesting the beneficial effects of vitamin D in various diseases. A literature search was carried out in PubMed for cases reporting vitamin D intoxication and overdose. Thirteen articles were included in this review. Intoxication was severe in the reported cases. Patients presented with serum vitamin D concentrations ranging between 150 and 1220 ng/mL and serum calcium concentrations between 11.1 and 23.1 mg/dL. Most of the reported patients showed symptoms of vitamin D toxicity such as vomiting, dehydration, pain, and loss of appetite. The underlying causes included manufacturing errors, overdosing by patients or prescribers, and combinations of these factors. Our literature search highlights the fact that even though vitamin D intoxication is rare, it does occur and therefore patients and prescribers should be more cognizant of the potential dangers of vitamin D overdose.

## 1. Introduction

Over the past two decades, interest in vitamin D has increased significantly. Besides playing important roles in calcium homeostasis and bone mineralization, vitamin D is now recognized as playing a role in the immune system, cardiovascular health, and cancer prevention [1,2,3]. Based on guidelines by the Institute of Medicine, recommended dietary allowances for vitamin D are 600 IU/day for those aged between 1 and 70 years and 800 IU/day for those aged 71 and above [4]. For babies below one year of age, the American Academy of Pediatrics recommends 400 IU/day [5]. These dietary recommendations should result in serum 25-hydroxyvitamin D levels of ≥20 ng/mL. At the other end of the scale, the Food and Nutrition Board has set upper intake levels of vitamin D at 2000 IU/day (50 μg/day) [6]. Doses higher than 50,000 IU/day raise serum concentrations above 150 ng/mL, leading to hypercalcemia [7]. 

With vitamin D deficiency being implicated in an increasing number of diseases [2,4,8,9], requests for serum vitamin D concentration measurements increased between the year 2000 and 2010 by over 80-fold [10]. The total 25-hydroxyvitamin D (25(OH)D) level, which includes both exogenous and endogenous vitamin D, is an appropriate indicator of the vitamin D that the body stores. Although there is no universal consensus about a treatment cut-off level, studies suggest 25 to 35 ng/mL as the minimum concentration of 25(OH)D needed to avoid adverse effects of deficiency, a problem particularly common at high latitudes, especially in the winter [11,12]. Suggestions to maintain vitamin D sufficiency include exposure to sunlight (the skin produces up to 10,000 IU/day after exposure to UV light), fortified foods (e.g., milk is fortified with 100 IU/cup), and over-the-counter supplementation (1000–2000 U/day). In addition, vitamin D is mostly found in fatty fish (447–1360 IU/100 g) and mushrooms [13,14]. However, vitamin D treatment is not without risks, as vitamin D toxicity has potentially serious consequences [15,16]. Due to a wide therapeutic index, vitamin D overdose is rare, but it does occur at excessively high doses. Vitamin D intoxication can be iatrogenic due to manufacturing errors or self-administration. In this report, cases of patients who had received high doses of vitamin D were reviewed and presented to describe features of vitamin D toxicity. 

## 2. Materials and Methods 

This review is based on a Pubmed search for articles published between 1936 and 2018 that described cases of vitamin D toxicity. The following terms were included in the search: ‘vitamin D toxicity cases’, ‘vitamin D intoxication cases’, and ‘vitamin D overdose cases’. These criteria identified 85 publications. After examining the title, abstract, and laboratory findings, 13 publications which reported the amount of vitamin D intake, serum vitamin D concentration upon admission, and a complete medical evaluation of the patient were included in the final analysis. Publications that were rejected included review papers (*n* = 21), animal studies (*n* = 16), cases not related to vitamin D intoxication (*n* = 29), cases of malignancy-associated hypercalcemia (*n* = 5), and a case of supposed vitamin D toxicity (*n* = 1) without any convincing laboratory data to support this diagnosis.

The Advanced Cohort Explorer (ACE) was used to generate the number of vitamin D tests ordered from the Mayo Clinic in Rochester, MN, USA between 1994 and 2017. The ACE is a clinical data repository maintained by the Unified Data Platform which contains patient information such as patient demographics, diagnoses, laboratory tests, and clinical notes. Data was obtained from multiple clinical and hospital source systems within the Mayo Clinic Rochester.

## 3. Results and discussion

**Figure 1:**
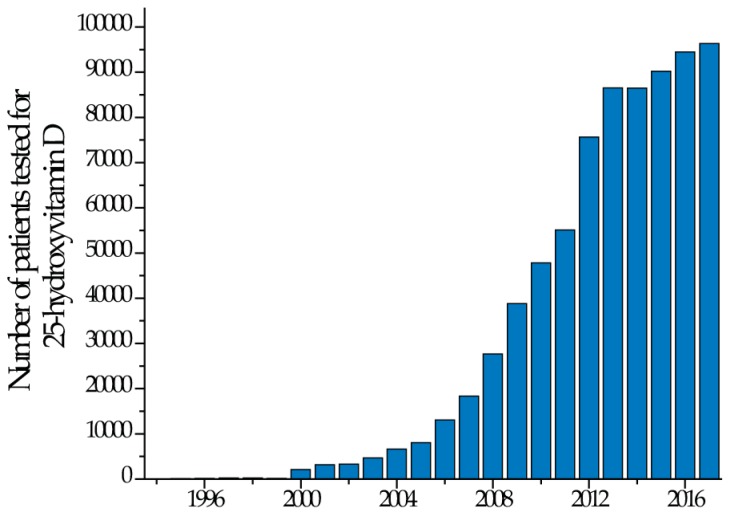
Sustained increase in testing volumes of vitamin D at the Mayo Clinic.

In the last two decades, many laboratories have experienced a surge in demand for vitamin D testing (Figure 1).

As more patients are tested, an increasing number of individuals with elevated serum 25(OH)D levels have been identified. Vitamin D toxicity occurs from exposure to extremely high doses of vitamin D supplementation, which can be the result of manufacturing errors or accidental or intentional incorrect dosing [15,17]. It is important to emphasize that supplementation is usually driven more by patients than physicians. Since vitamin D increases calcium absorption in the gastrointestinal tract, vitamin D intoxication manifests primarily as hypercalcemia and hypercalciuria. This leads potentially to muscle weakness, hypertension, neuropsychiatric disturbances, gastrointestinal upset, polyuria and polydipsia, renal calculi, and, in extreme cases, renal failure, deposition of calcium phosphate crystals in soft tissues throughout the body, cardiac arrhythmias (reduced action potential), calcification of coronary vessels and heart valves, and ultimately, death [18,19]. 

Various cut-off values for the upper levels of serum vitamin D are used across the USA [4,20,21]. However, the patients listed in Table 1 had levels above all cut-off values in common use. The table summarizes the presented cases and includes patients’ age, vitamin D dose, vitamin D test results, and total calcium levels. 

### 3.1. Manufacturing Errors

Vitamin D intoxication with severe hypercalcemia has been reported in adult and pediatric cases due to manufacturing errors of dietary supplements and are summarized in Table 1. Anik et al. and Kara et al. described 10 children aged between one and four years who were admitted to the hospital with symptoms of abdominal pain, vomiting, dehydration, and poor appetite [22,23]. In two children, renal ultrasonography showed the development of bilteral nephrocalcinosis. Laboratory findings showed total calcium results between 13.4 and 19.4 mg/dL (normal range 8.4–10.2 mg/dL) and serum 25(OH)D between 340 and 962 ng/mL (normal range 25–80 ng/mL). The medical history and conversations with the parents revealed that all children were supposed to be receiving 200 IU of vitamin D each day for up to one month as a dietary supplement to improve appetite. Laboratory analysis of the supplements showed that the actual vitamin D content was 4000 times the labeled concentration. As a result, the children had been ingesting between 266,000 and 800,000 IU each day without any investigation for a specific diagnosis.

Araki et al. reported two adult cases of vitamin D intoxication with dietary supplements due to manufacturing errors that resulted in 1000-fold higher levels of vitamin D [24]. Laboratory findings in the patients upon admission showed 25(OH)D levels between 654 and 1220 ng/mL (normal range 30–80 ng/mL) and total calcium between 13.2 and 15.0 mg/dL (normal range 8.4–10.2 mg/dL).

Koutkia et al. described a 42-year-old male who presented with classic symptoms of hypercalcemia, serum 25(OH)D levels of 487.3 ng/mL, and total calcium of 15.0 mg/dL [25]. Analysis of the supplement by HPLC revealed that the actual dose that the patient had been taking was 78–1302 times the recommended upper limit of 2000 IU/d.

### 3.2. Inappropriate Administration

In 2015, Ketha et al. reported a pediatric case of vitamin D intoxication with vitamin D supplements [26]. An infant was admitted to the hospital with significant dehydration, vomiting, constipation, lethargy, and weight loss. Routine biochemical evaluation demonstrated total calcium levels of 18.7 mg/dL, indicating severe hypercalcemia. Vitamin D metabolites were tested in the infant’s blood and were as follows: 25(OH)D_3_, 293 ng/mL (optimal range of total 25(OH)D: 20–50 ng/mL); and 1,25(OH)_2_D_3_, 138 pg/mL (optimal range: 24–86 pg/mL). Renal ultrasonography revealed nephrocalcinosis. A detailed discussion with the mother revealed that the infant was receiving 50,000 IU of vitamin D per day, while the recommended dose on the label was 2000 IU. 

Two other case reports of breastfed infants who had vitamin D toxicity were described by Bilbao [27]. The two infants (aged 2.5 months and 3.5 months, respectively) were admitted to the hospital with an identical presentation, including decreased feeding, lethargy, moderate dehydration, and inconsolable crying. Both infants were exclusively breastfed and had been receiving over-the-counter vitamin D supplementation. During the discussion with the parents, it was revealed that the infants had received vitamin D supplementation far above the recommended dose, resulting in hypervitaminosis D (25(OH)D: 644–680 ng/mL) and hypercalcemia (total calcium: 15–21 mg/dL).

Rocha and Santos reported the case of a 19-year-old male who used a parenteral formulation of vitamins A, D, and E restricted to veterinary use (5,000,000 IU of vitamin D_3_ in a 100-mL vial) [28]. The patient was admitted to the hospital with a three-week history of anorexia, nausea, and vomiting. Serum 25(OH)D was found to be 150 ng/mL and total calcium was found to be 14.8 mg/dL. He later admitted taking 300 mL of the product over one year in order to give the impression of bigger muscles through the swelling of the tissue after injecting the oily formulation. Treatment of the patient included normal saline, furosemide, and zoledronic acid that rapidly normalized calcium levels and renal function.

### 3.3. Incorrect Prescribing by Physicians

Physicians treat patients with high doses of vitamin D for various disorders and in some instances, prescribe doses that exceed the suggested recommendations. Kaur et al. and Koul et al. described patients who were prescribed high doses of vitamin D supplements for various ailments [29,30]. Patients with ages ranging from 42 to 86 years presented with clinical features of vitamin D overdose: lethargy (*n* = 3), vomiting (*n* = 9), polyuria (*n* = 5), polydipsia (*n* = 5), altered sensorium (*n* = 9), renal dysfunction (*n* = 5), weight loss (*n* = 5), nausea (*n* = 5), and constipation (*n* = 4). Laboratory tests revealed hypercalcemia in all of these patients, with serum calcium level between 11.0 and 15.7 mg/dL, serum phosphates ranging from 2.0 to 8.6 mg/dL (normal range 3.5–5.0), and serum 25(OH)D between 164 and 1161 ng/mL. Differential diagnosis of hypercalcemia included multiple myeloma, granulomatous disease, renal disease, and hyperparathyroidism, but based on further evaluations, these diseases were ruled out. It was determined that the patients had taken 2,220,000–60,000,000 IU of vitamin D over a period of 4–7 weeks for various conditions that included back pain (*n* = 4), radiculopathy (*n* = 2), osteoarthritis (*n* = 2), or generalized weakness (*n* = 2). These megadoses resulted in vitamin D toxicity in all patients. Treatment of hypercalcemia included steroids, bisphosphonates, and calcitonin. Eventually clinical recovery of these patients was observed.

Bansal also described an iatrogenic case of hypervitaminosis D [31]. It involved a 45-year-old female who had received a total of 6,000,000 IU of vitamin D as an intramuscular injection within a period of two weeks. The patient was admitted to the hospital with a history of recurrent vomiting, pain in her abdomen, polydipsia, anorexia, and constipation over the previous 1.5 months. Laboratory results revealed 25(OH)D and total calcium levels to be 150 ng/mL and 23.1 mg/dL, respectively. It was later revealed that the intramuscular injection had been prescribed after her knee surgery. The hypercalcemia was treated with intravenous fluid, diuretics, and calcitonin, and the high serum calcium normalized within 15 days.

Iatrogenic hypervitaminosis D was reported in elderly subjects in whom toxicity occurred due to excessive administration of vitamin D by oral and intramuscular routes [32]. The injections of vitamin D (600,000 IU/injection) were prescribed by the physician to improve health and to reduce the frailty of the elderly. Fifteen patients aged between 42 and 85 years presented to the hospital with elevated serum calcium and serum 25(OH)D levels. Median (range) serum 25(OH)D levels and median (range) total serum calcium levels were 118.1 (103–164) ng/mL and 13 (10.9–15.1) mg/dL, respectively. Clinical symptoms upon admission included altered sensorium (82.4%), dehydration (88.2%), vomiting (35.3%), anorexia (70.6%), fatigue (82.4%), generalized body weakness (88.2%), constipation (52.9%), polyuria (76.5%), and polydipsia (70.6%). Among the 15 cases, 12 showed evidence of renal dysfunction.

Chowdry reported on 19 patients aged between 45 and 89 years with vitamin D toxicity-induced acute kidney injury (AKI) [33]. Clinical manifestations upon admission to the hospital included nausea and vomiting (*n* = 11), altered sensorium (*n* = 7), constipation (*n* = 9), acute pancreatitis (*n* = 2), AKI (*n* = 16), acute chronic kidney disease (*n* = 3), and weight loss (*n* = 2). The median serum calcium level was 13.0 mg/dL, with the lowest concentration being 11.9 mg/dl and highest concentration of 15.2 mg/dl. The median serum 25(OH)D concentration was 371 ng/ml, with the lowest concentration being 190 ng/ml and highest concentration being 988 ng/ml. Enteral or parenteral overcorrection of vitamin D deficiency was the cause of vitamin D toxicity-induced AKI in all cases. All the patients were prescribed vitamin D by their physician for various ailments: bone pains (*n* = 6), generalized aches and pains (*n* = 6), fatigue (*n* = 5), and myalgia (*n* = 2). Management of AKI and hypercalcemia for all patients included intravenous fluids, diuretics, calcitonin, and glucocorticoids. 

## 4. Conclusions

Vitamin D plays an important role in calcium homeostasis and bone mineralization. Vitamin D also exhibits many non-skeletal effects, particularly in cancer, cardiovascular diseases, and autoimmune diseases. However, inappropriate use of vitamin D supplements can lead to toxicity and life-threating hypercalcemia. Due to a wide therapeutic index, vitamin D toxicity is extremely rare; however, it does occur at excessively high doses. The highest daily intake of vitamin D that will pose no risk of adverse effects is not known. The current allowable upper intake of vitamin D for long-term supplementation is 2000 IU/day. Here, we show cases where vitamin D toxicity was caused by formulation, administration, subscription errors, which resulted in excessive dosing. The vitamin D doses reported in these cases ranged from 50,000 IU/day to 2,604,000 IU/day and led to marked hypercalcemia (total calcium between 11.1 and 23.08 mg/dL) and serum 25(OH)D concentrations greater than 150 ng/mL. The clinical manifestations of vitamin D toxicity reported in these cases were a consequence of the hypercalcemia and included nausea, vomiting, weakness, polyuria, nephrocalcinosis, and renal failure. This indicates that 25(OH)D levels above 150 ng/mL are likely to be associated with toxicity and should be avoided at all costs.

We recommend that more conventional doses of vitamin D supplements, in line with Institute of Medicine and other published guidelines, should be used, unless there is an urgent need for rapid increases in 25(OH)D levels. In the case of high-dose treatment, extra care should be taken in instructing patients about proper use; they should be instructed to avoid additional over-the-counter supplements, and the number of high doses prescribed should be limited, with 25(OH)D levels being checked regularly.

With regards to over-the-counter supplements, patients should be advised of current dosing recommendations, in particular that 2000 IU/day should not be exceeded in the long term without prior consultation with a physician. They should also be instructed as to symptoms of hypercalcemia and to cease taking supplements if such symptoms occur and have their serum calcium, phosphate, and 25(OH)D levels measured before resuming supplementation.

Finally, while relatively uncommon, vitamin D intoxication should always be considered as a differential diagnosis when evaluating patients with hypercalcemia.

## Figures and Tables

**Table 1:** Test results and dosage information of patients with vitamin D toxicity. AKI: acute kidney injury.

Age (years)	Vitamin D dose	Form of Intake	Reason	Vitamin D, Serum (ng/m)	Total Ca, Serum (mg/dL)	Symptoms	Ref.
0.4–4.2 (*n* = 7)	260,000–800,000 IU/day	Fish oil supplements	Labeling errors	340–962	13.4–18.8	Weakness, loss of appetite, vomiting	[23]
1–2 (*n* = 2) 1	200 IU/day (2–4weeks) 200 IU/day (1 month)	Oral preparation Oral preparation	Labeling errors Labeling errors	>160 760	13.7–19.3 19.4	Abdominal pain, vomiting Poor appetite, vomiting	[22]
58 40	1,864,000 IU (2 months) 970,000 IU (1 month)	Oral supplements Oral supplements	Labeling errors Labeling errors	1220 645	15 13.2	Fatigue, thirst, polyuria Nausea, vomiting, thirst, polyuria, muscle aches	[24]
42	156,000–2,604,000 IU/day (2 years)	Oral supplements	Labeling errors	487.3	15	Dehydration, fatigue, loss of apetite	[25]
0.3	50,000 IU/day (2 months)	Oral supplements	Inappropriate administration	294	18.7	Vomiting, diarrhea, dehydration	[26]
0.3–0.2 (*n* = 2)	20,000 IU/day (1.5 weeks)	Oral supplements	Inappropriate administration	644 680	15 L 21 L	Poor appetite, lethargy, crying	[27]
19	15,000,000 IU (1 year)	Injection	Inappropriate administration	150	14.8	Anorexia, nausea, vomiting	[28]
42–86 (*n* = 16)	2,220,000–6,360,000 IU (1–3 months)	Injection or oral sachets	Iatrogenic (body aches and fatigue)	175–1161	11.1–15.7	Nausea, vomiting, constipation	[29]
48–75 (*n* = 0)	3,000,000–60,000,000 IU (1–4 months)	Injection or oral sachets	Iatrogenic (various indications)	164–306	12–13.98	Vomiting, polyuria, anorexia	[30]
45	6,000,000 IU (2 weeks)	Injection	Iatrogenic (knee surgery)	150	23.1	Anorexia, vomiting, abdominal pain	[31]
42–85 (*n* = 15)	600,000 IU (1 month–3 years)	Oral supplements + injections	Iatrogenic (improve health)	103–164	10.9–15.2	Altered sensorium, dehydration, vomiting, anorexia	[32]
45–89 (*n* = 19)	4,200,000–9,000,000 IU (1–5 months)	Oral tablets or injections	Iatrogenic (bone pain, aches, fatigue)	190–988	11.9–15.2	Vomiting, altered sensorium, AKI, constipation,	[33]
75	50,000 IU/day (1 year)	Oral supplements	Iatrogenic (hypoparathyroidism)	243	15.3	Altered mental status	[34]
